# Effects of Daily Ingestion of Two SunGold Kiwifruit for 6 Weeks on Metabolic and Inflammatory Biomarkers: A Randomized, Cross-Over, Exploratory Intervention Study

**DOI:** 10.3390/foods12234236

**Published:** 2023-11-23

**Authors:** Suman Mishra, Kerry Bentley-Hewitt, Tony McGhie, Karl Fraser, Duncan Hedderley, Sheridan Martell, Hannah Dinnan, John Monro

**Affiliations:** 1 The New Zealand Institute for Plant and Food Research Limited, Palmerston North 4442, New Zealand; suman.mishra@plantandfood.co.nz (S.M.); kerry.bentley-hewitt@plantandfood.co.nz (K.B.-H.); tony.mcghie@plantandfood.co.nz (T.M.); duncan.hedderley@plantandfood.co.nz (D.H.); sheridan.martell@plantandfood.co.nz (S.M.); hannah.dinnan@plantandfood.co.nz (H.D.); 2 AgResearch Limited, Palmerston North 4410, New Zealand; karl.fraser@agresearch.co.nz; 3 Riddet Institute, University Avenue, Fitzherbert, Palmerston North 4474, New Zealand

**Keywords:** kiwifruit, metabolic health, inflammation, vitamin C, plasma lipids, C-reactive protein, plasma short-chain fatty acids

## Abstract

Kiwifruit contain many components, some considered beneficial, such as vitamins, phytochemicals and dietary fibre, and others potentially harmful, such as fructose and glucose in fruit sugars. In a 6-week, randomised, crossover study aimed at exploring the net effects of daily consumption of kiwifruit, 23 healthy participants consumed two *Actinidia chinensis* var. *chinensis* ‘Zesy002’ (marketed as Zespri™ SunGold™ Kiwifruit) per day as part of their customary diet (intervention) or without kiwifruit (control) as their customary diet for 6 weeks in a cross-over study. Anthropometric data, venous blood, and urine samples were collected at the start and end of the 6-week intervention and control periods for the measurement of physical changes, plasma glucose, insulin, glycated haemoglobin, short-chain fatty acids, blood lipids, uric acid, inflammatory biomarkers, and urinary ascorbic acid. Variables were measured between the start and finish of interventions, and between intervention and control periods. Food diaries were completed on the 3 days before blood sampling to estimate dietary ascorbic acid and dietary fibre intakes. Despite urinary vitamin C and food diaries indicating compliance, and good precision in measurements, there were no appreciable changes in biomarkers during the study, either within or between intervention and control periods, that would indicate a change in health status. Thus, the sizes of any effects of kiwifruit ingestion were too small to become significant under the test conditions used, indicating a high probability that daily ingestion of two SunGold kiwifruit is safe with respect to metabolic health.

## 1. Introduction

Fruit are generally regarded as part of a natural, healthy, mixed diet, with epidemiological evidence indicating an optimal intake of five servings per day [1], which is the current US recommendation. Kiwifruit are an important export fruit, promoted for their multiple health benefits [2]. However, like most ripe fruit they are high in fruit sugars, including fructose and glucose (New Zealand Food Composition Database, www.foodcomposition.co.nz, accessed 31 March 2023). Therefore, increasing kiwifruit consumption could increase net intake of fruit sugars in the unconstrained (non-carbohydrate exchange) ad libitum diet of free-living humans, which could be of concern in populations that already have a high intake of carbohydrates. With current anxiety regarding the global epidemic of glucose intolerance [3], and associated medical complications that result from chronic exposure to highly reactive ketoses in the body [4,5], there is a risk of consumers becoming reluctant to consume fruit to avoid high-blood sugar responses. If such reluctance translates to consumer resistance to fruit, market demand could suffer while consumers fail to obtain the health benefits of fruit.

**Figure A1:**
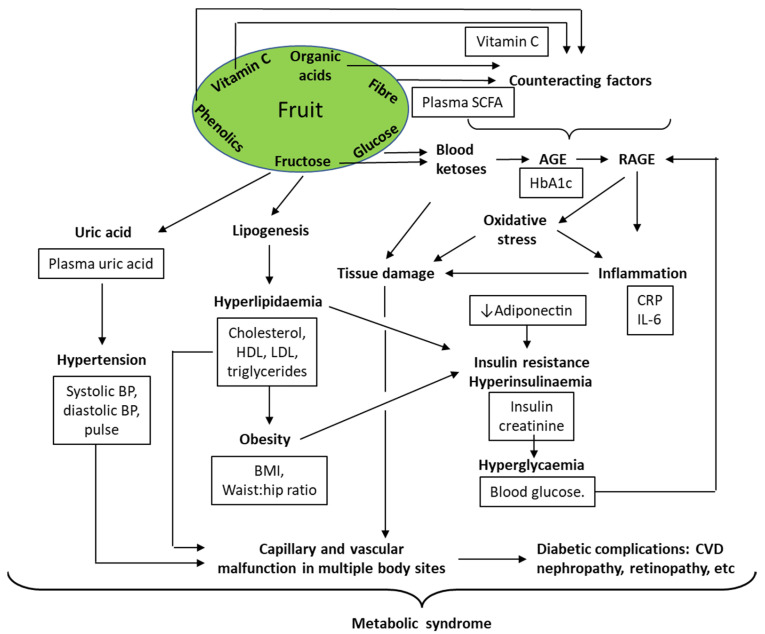
Variables measured in 6-week kiwifruit intervention study (non-bold in boxes) and their relationship with possible physiological and metabolic effects of kiwifruit components (bold, unboxed). Abbreviations: AGE, advanced glycation end product; BMI, body mass index; BP, blood pressure; CRP, C-reactive protein; CVD, cardiovascular disease; RAGE, AGE receptor; HbA1c, glycated haemoglobin; HDL, high density lipoprotein, LDL, low density lipoprotein; IL-6, interleukin 6.

Therefore, irrespective of the epidemiological findings that fruit as a food category is negatively related to all-cause mortality [6], and that many studies of fructose effects have used higher doses than would occur in fruit [7,8], it is prudent from the health and marketing perspective to scientifically confirm that kiwifruit specifically does not contribute to risk of metabolic syndrome, or the cluster of conditions it embraces (Figure A1).

Fruit sugars may be harmful through several post-absorptive mechanisms, some related to postprandial glycaemia, and others to the high proportion of fructose in fruit sugars. Two kiwifruit can cause a definite and significant postprandial elevation in blood glucose in healthy individuals [9], while fructose consumption is associated with elevated postprandial triglycerides [10]. Furthermore, glucose and fructose may have a synergistic effect on risk factors for cardiovascular disease in young adults [11].

The mechanisms through which fruit sugars may be harmful include the following:

- Creating insulin demand leading to B-cell exhaustion [12].
- Reacting chemically and non-specifically with various body components to form advanced glycation end (AGE) products, which react with AGE receptors (RAGE) that induce inflammatory responses [4].
- Overloading the mitochondrial respiratory system, leading to formation of reactive oxygen species (ROS) and creating a state of oxidative stress, which is a further inflammatory stimulus [13].
- Causing fructose-stimulated lipogenesis leading to dyslipidaemia, which is associated with insulin resistance and increased cardiovascular disease risk [14,15] even in healthy individuals [10].
- Increasing uric acid production as a by-product of fructose catabolism, with possible elevation of blood pressure and other biomarkers of metabolic syndrome [16].

While ripe fruit may be high in fruit sugars, they may also contain compounds that counteract the possibly damaging effects of high postprandial blood sugar concentrations. These include the following:

- Components that slow the absorption of sugars so that sugar disposal processes for removal of fructose and glucose from the blood can match blood sugar loading, preventing high and/or prolonged blood sugar concentrations. Cell wall remnants (dietary fibre), organic acids, and phenolic compounds may all slow glucose absorption from the gut [17].
- Antioxidant phytochemicals, including phenolic compounds from fruit, may augment intrinsic antioxidant systems such as those based on reduced glutathione (GSH) [18].
- Cell wall remnants that are fermented in the hind gut to microbial metabolites such as short-chain fatty acids (SCFA), which may have multiple effects on biochemical systems mediating the systemic damage that emerges from long-term hyperglycaemia [19].

Kiwifruit is noted for its high content of ascorbic acid [2], which may act as an antioxidant. However, even vitamin C may be pro-oxidant under certain conditions, and reactive products of vitamin C catabolism may cause protein damage by dehydroascorbylation and glycation [20].

With such an array of counterpoised factors that may positively and/or negatively affect biomarkers of health in response to fruit ingestion, it is difficult to assess the net changes caused by any given fruit. Therefore, we have tested the effect of including two kiwifruit per day in a customary diet on a spectrum of metabolic and inflammatory biomarkers that might indicate a change in long-term disease risk in a healthy mixed New Zealand population. The trial complements two earlier studies of kiwifruit effects, both parallel group trials, one for 7 weeks with Asian participants (genetically predisposed to glucose intolerance) [21] and the other for 12 weeks with prediabetic participants (metabolically predisposed to glucose intolerance) [22]. The present study used a cross-over design in which two kiwifruit per day (200 g edible portion) were ingested for 6 weeks with kiwifruit and no-kiwifruit periods running in parallel. Therefore, in contrast to epidemiological studies on the association between fruit ingestion and health [6], the present study was well controlled, and the subjects were continually monitored. The participants formed a cohort from a healthy population, which may differ from a glucose-intolerant population in its postprandial response to dietary fructose [10].

Amongst the analyses were changes in plasma SCFA that might result from an altered load of fermentable dietary fibre in the colon. The dietary fibre (cell wall) component of kiwifruit is present predominantly in the form of fruit parenchyma cell walls, which are largely fermented in the monogastric hind gut [23]. An intake of two kiwifruit per day may contribute 3–4 g of dietary fibre, and therefore make an appreciable addition to the fermentable fibre load in modern human diets, which are often deficient in dietary fibre.

Short-chain fatty acids from bacterial fermentation of dietary fibre are known to be absorbed from the colon into the portal vein and transported to the liver in humans. Beyond the liver a proportion of them are delivered to a range of tissues and organs of the periphery, where they may influence metabolic control, satiety, body composition, and immunity. The discovery of receptors for SCFA throughout the body suggests that they may play a role in metabolic regulation [24].

The aim of this study was to determine whether including two SunGold kiwifruit per day in the diets of healthy humans for 6 weeks would elicit changes in a range of metabolic and inflammatory biomarkers linked to long-term health outcomes (Table A1) and encompassed by metabolic syndrome (Figure A1). In metabolically healthy individuals, in whom homeostasis is functioning normally, it may not be sufficient to detect longer-term effects of blood glucose fluctuations on inflammatory and metabolic markers, not because they are non-existent, but because the effect sizes may be so small in healthy people that a large cohort would be required to detect them. In the present study, we did not attempt to reject a null hypothesis that there are no effects, with the risk of type 2 error, but instead test whether any effect sizes are large enough to emerge under the experimental conditions used. If they are not, a moderate intake of kiwifruit in a mixed diet can reasonably be regarded as having a low probability of contributing to risk of metabolic disorder in healthy individuals.

## 2. Materials and Methods

Export quality SunGold kiwifruit, *Actinidia chinensis* var. *chinensis* ‘Zesy002’ (marketed as Zespri™ SunGold™ Kiwifruit) were supplied by Zespri International Limited (Tauranga, New Zealand) as required over the course of the trial.

### 2.1. Experimental Design

**Figure 1:**
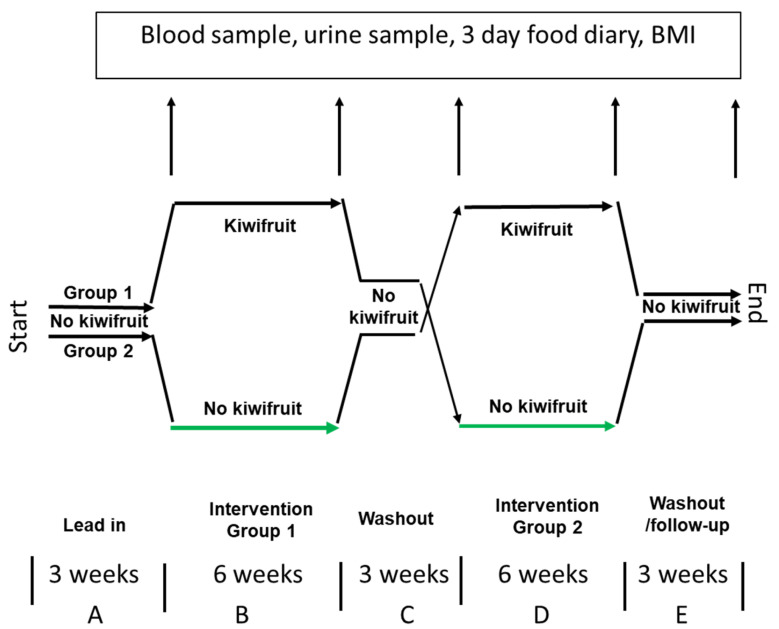
Design of crossover study of the effects of ingesting two kiwifruit per day for 6 weeks. BMI = body mass index.

A total of 24 participants were recruited and randomly allocated to two treatments, control (n = 12) and kiwifruit intervention (n = 12), running in parallel for 6 weeks, with the groups crossed over after a 3-week washout period. The intervention periods were preceded by a 3-week kiwifruit-free lead-in and followed by a 3-week kiwifruit-free follow-up at the completion of the trial, which therefore ran for 21 weeks (Figure 1).

### 2.2. Participant Number (n)

**Figure A2:**
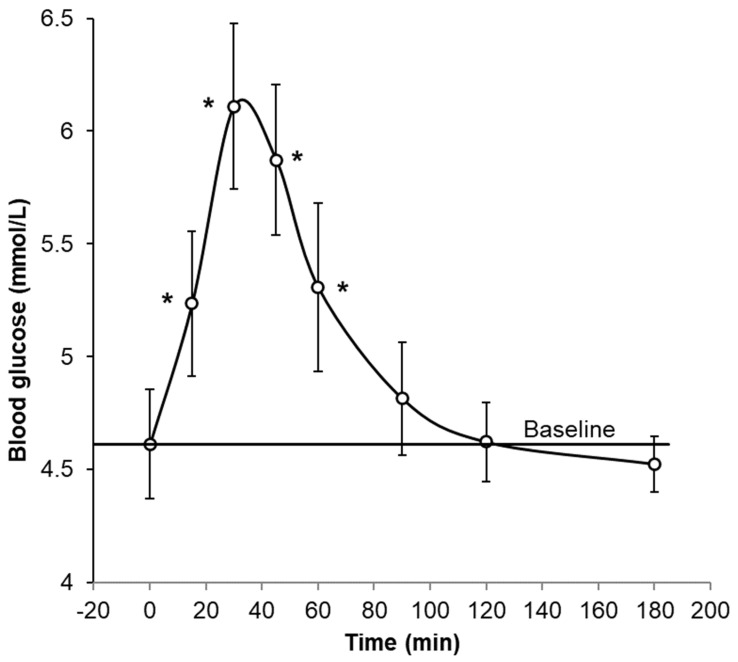
Two SunGold kiwifruit (200 g) alone had a moderate effect size, raising blood glucose concentrations significantly above baseline in healthy participants fed at breakfast time after an overnight fast (*p* < 0.001, n = 21). Means ± SEM. Points significantly higher than baseline are shown with an asterisk (LSD 0.38; Dunnett’s test). Blood glucose concentrations were monitored by capillary finger-prick analysis.

The sample size of n = 24 was adequate to detect a significant effect of the 200 g KF (kiwifruit) dose used in the present study on blood glucose concentrations (Figure A2). Comparable group sizes have allowed for significant effects of dietary fibres on plasma lipids and glucose responses [25], and effects of fatty acids on inflammatory markers in blood [26], to be shown, while similar numbers (n = 27) were used to show acute effects of fructose on glucose, insulin, and triglyceride levels after a solid meal in a crossover study [27]. In the present study, practicalities and resources limited the sample size and the number of outcome variables, although the n was large enough to detect any substantial changes, and failure to achieve statistical significance would indicate a small effect size, if any.

### 2.3. Participants

A total of 24 participants were recruited, online and by newspaper advertisement in which the study was briefly described. Volunteers were pre-screened by the staff from The New Zealand Institute for Plant and Food Research Limited (Plant & Food Research, Palmerston North, New Zealand) who described the nature of the study and the involvement and responsibilities of participants and assessed eligibility to take part. Eligible volunteers were invited to attend the Plant & Food Research clinic. Their blood glucose and glycosylated haemoglobin (HbA1c) concentrations were measured using a finger-prick capillary blood sample to make sure they were within the normal range. Each volunteer was given an informed consent form and an information sheet containing study details. The participants received a small remuneration for taking part in the study in the form of a NZ$20 supermarket voucher at the initial screening and a NZ$20 voucher for each testing session.

The 24 participants were randomly assigned to the initial intervention and control arms of the trial. Criteria for participant selection were as follows:

### 2.4. Inclusion Criteria

Age: Between 25 and 60 years.

Gender: Male

Glucose tolerance: No history of diabetes (type 1 or 2) or evidence of glucose intolerance in a preliminary oral glucose tolerance screening test using Diabetes New Zealand guidelines: fasting blood glucose < 6.0 mmol/L, HbA1c < 41 mmol/mol.

Body Mass Index (BMI): Between 20 and 37.5 kg/m^2^.

Health: Healthy as gauged by self-assessment using the General Health Questionnaire.

Agreement: Written informed consent to comply with the conditions of the trial.

### 2.5. Exclusion Criteria

Glucose intolerance: Any history of diabetes (Type 1 or 2) or evidence of glucose intolerance in a preliminary test using Diabetes New Zealand guidelines: Fasting blood glucose < 6.0 mmol/L, HbA1c < 41 mmol/mol.

Intolerance of kiwifruit.

Any illness or gastrointestinal disorder within the 3 weeks prior to the trial.

### 2.6. Participant Instructions

Participants were assigned randomly to two groups of 12 subjects each. The first group was instructed to continue with their customary diet for 12 weeks (3 weeks lead-in, 6 weeks control, 3 weeks washout) after which they entered the kiwifruit intervention phase during which two kiwifruit per day were ingested for 6 weeks followed by a 3-week washout. The second group consumed no kiwifruit for 3 weeks (lead-in) and then consumed two SunGold kiwifruit per day for 6 weeks (intervention), after which they consumed no kiwifruit for 12 weeks (3 weeks washout, 6 weeks control, 3 weeks follow up) (Figure 1). Both groups were asked to not take vitamin supplements during the course of the trial, but to otherwise consume their normal diets. During the lead-in, control, washout, and follow-up periods all subjects were allowed to return to their customary diets with the exclusion of kiwifruit and vitamin supplements.

Participants were instructed to attend the Plant & Food Research clinic in the mornings at the end of the 3rd, 9th, 12th 18th, and 21st weeks of the trial to provide blood and urine samples for analysis. They were therefore expected to attend the clinic five times during the trial (Figure 1).

Participants were also asked to fill out a 3-day food diary five times, covering the 3 days leading up to each the of the five visits to the clinic. The diet record asked participants to record the time of ingestion, a description of the food or drink consumed (such as variety, brand, cut of meat), the food or beverage preparation method, and the amount consumed Weight or standard measure). The food diaries were used to estimate intakes of vitamin C and dietary fibre.

### 2.7. Blood and Urine Sampling

On five visits to the clinic, the participants were asked to provide fasting venous blood samples, which were taken by a trained phlebotomist. The subjects were seated and once relaxed and comfortable venous blood was withdrawn from the anterior cubital fossa into three 10 mL Vacutainer™ tubes. One tube was centrifuged immediately (1000× *g*, 15 min, 4 °C) and required plasma aliquots pipetted into cryotubes to be immediately stored at −80 °C until analysis. A second tube was allowed to stand at room temperature (20 °C, 1 h), centrifuged (2000× *g*, 15 min, 4 °C), and the serum aliquots for analysis were pipetted into cryotubes for immediate storage at −80 °C until analysis. A third blood sample was delivered immediately to the medical laboratory for creatinine analysis.

A urine sample was requested and supplied in a 60 mL screw-capped specimen bottle at the time of blood collection.

### 2.8. Analyses

A summary of the relevance of the biomarkers measured is given in Table A1.

#### 2.8.1. Plasma Ascorbic Acid Analysis

For plasma ascorbic acid analysis, venous blood was collected in EDTA vacutainer tubes and immediately centrifuged at 1000× *g* for 15 min at 4 °C. The plasma supernatant was removed into a chilled Eppendorf tube and a 0.2 mL aliquot was removed and added to an equal volume of 10% metaphosphoric acid in 2 mM EDTA solution, vortex mixed, and rested on ice for 5 min before spinning at 16,000× *g* for 10 min at 4 °C. The supernatant was removed and stored at −80 °C until analysis by ultra-high-performance liquid chromatography coupled with mass spectrometry (UHPLC-MS). For analysis by UHPLC-MS the samples were thawed in ice, diluted with equal volumes of acetonitrile/MilliQ water (90/10) in microcentrifuge tubes, mixed immediately by inversion, vortexed, and centrifuged for 15 min at 4 °C and 16,000× *g*. A 0.2 mL aliquot of supernatant was transferred to HPLC vials for analysis by UHPLC-MS.

#### 2.8.2. Urine Ascorbic Acid Analysis

Urine ascorbic acid was measured at the end of the no-kiwifruit and kiwifruit intervention periods. Urine specimens were collected into 60 mL specimen jars and placed in darkness on ice until a 0.5 mL aliquot was added to an equal volume of 10% metaphosphoric acid in 2 mM EDTA solution, vortex mixed, and stored at −80 °C until analysis by HPLC with UV/visible detection. As for plasma, the HPLC the samples were thawed on ice and a 0.2 mL sample was added to 0.4 mL of acetonitrile in microcentrifuge tubes, mixed immediately by inversion, vortexed, and centrifuged for 15 min at 4 °C and 16,000× *g*. A 0.2 mL aliquot of supernatant was transferred to vials for HPLC analysis using UV/visible detection.

#### 2.8.3. Dietary Fibre and Ascorbic Acid Intakes

Dietary fibre and ascorbic acid intakes were estimated from the 3-day food diaries of the kiwifruit and non-kiwifruit groups over the 3 days prior to blood sampling, using FoodWorks software (Xyris Software (Australia) Pty Ltd., Brisbane, Australia). The change in intakes between the no-kiwifruit and kiwifruit periods was calculated for each participant.

#### 2.8.4. Anthropometric Changes

BMI was calculated from weights measured using a Seca 813 digital floor scale and heights measured with a Seca 217 stadiometer (Seca gmbh & Co., Hamburg, Germany).

Waist:hip ratio was determined from waist and hip girths measured with a Seca 201 tape measure (NZ Fitness Gear, Wellington, New Zealand).

#### 2.8.5. Cardiovascular Variables

Systolic and diastolic blood pressure as well as pulse were measured using an OMRON Digital Automatic Blood Pressure Monitor.

#### 2.8.6. Metabolic Biomarkers

In a preliminary study the 200 g KF dose given daily was shown to cause a significant postprandial increase over baseline in blood glucose when fed on its own to the number of subjects used in the present study (Figure A2).

Fasting blood glucose was measured in capillary finger prick blood samples using a HemoCue Glucose 201 DM RT Analyser. HbA1c was measured using a HemoCue HbA1c 501 Analyser, with a capillary finger prick sample also.

Creatinine and uric acid were measured by routine methods for human blood samples in a certified medical laboratory (Medlab Central, Palmerston North, New Zealand).

#### 2.8.7. Hormones and Peptides

Insulin, adiponectin, C-reactive protein (CRP), and plasma interleukin 6 (IL-6) were determined by ELISA assay using the following kits and the following manufacturer’s instructions: Human Insulin ELISA (Millipore Cat. EZHI-14K, Burlington, MA, USA), Human Adiponectin Platinum ELISA (Invitrogen Cat. BMS2032/2, Waltham, MA, USA); Human CRP ELISA (antibodies-online, Inc., Pottstown, PA, USA, Cat. ABIN366539); Human IL-6 High Sensitivity ELISA (Invitrogen Cat. BMS213HS).

#### 2.8.8. Plasma Lipids

Plasma lipids—total cholesterol (mmol/L), low-density lipoprotein (LDL)-cholesterol (mmol/L), high-density lipoprotein (HDL)-cholesterol (mmol/L), and triglycerides (mmol/L)—were determined by routine methods in a certified analytical laboratory (Nutrition Laboratory, Massey University, Palmerston North, New Zealand).

#### 2.8.9. Plasma Short-Chain Fatty Acids

For plasma preparation, the tubes containing blood samples were cooled in salt/ice and centrifuged (1000× *g*, 15 min, 4 °C). For metabolomic SCFA analysis, a 0.5 mL sample of supernatant (plasma) was immediately frozen and stored at −80 °C until SCFA analysis. Plasma SCFA analyses were conducted at AgResearch metabolomics facility (Palmerston North, New Zealand). The extraction and instrumental analysis was performed using the published method of Moreau et al. [28], which utilizes stable isotope internal standards, solid phase microextraction (SPME), and gas chromatography mass spectrometry (GC-MS) to measure the SCFAs.

### 2.9. Statistical Analysis

An intention to treat analysis (ITT) was carried out, with available data from all participants included in the statistical analysis. Analysis of variance (ANOVA) was used to investigate whether the treatments differed in their effects. Residuals were inspected as part of the analysis of variance. Most variables were normally distributed with similar variances; urinary vitamin C needed to be square-root transformed to stabilize variances. Phase (i.e., first or second treatment) and treatment were fitted as fixed factors; participant was fitted as a random factor. Two approaches were used to incorporate information about the readings at the start of the treatment period; in one the difference between final and start values was analysed, and in the other the final values were analysed but the corresponding start values were included as a covariate.

*p*-values ≤ 0.05 were considered statistically significant with other differences noted.

## 3. Results

### 3.1. Participant Characteristics

The characteristics of the study population are presented in Table A2. The final study population was predominantly Caucasian (n = 15) with one Chinese, one Vietnamese, and three Indian participants. To minimise the possible effects of monthly hormonal changes, only males were recruited. All participants were healthy with no indication of glucose intolerance based on fasting blood glucose and HbA1c measurements (Table A2).

**Figure A3:**
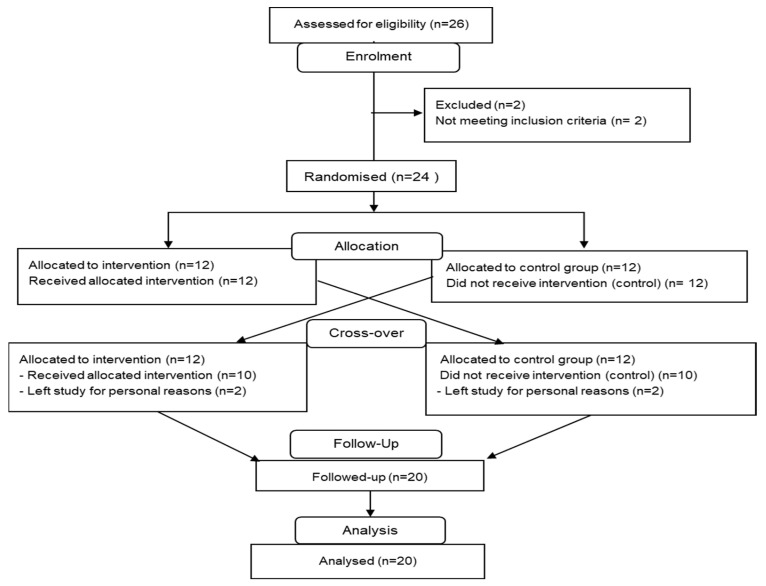
CONSORT diagram for the cross-over study of the effects of daily kiwifruit ingestion (intervention) compared with no-kiwifruit ingestion (control).

Most of the participants completed the full 21 weeks of the trial, most attended all five visits to the clinic, and most completed the 3-day food diaries as requested. Four participants withdrew because of changed life circumstances, none because of any reaction to the treatments given. The CONSORT diagram for the study is shown in Figure A3.

#### 3.1.1. Ascorbic Acid

**Figure 2:**
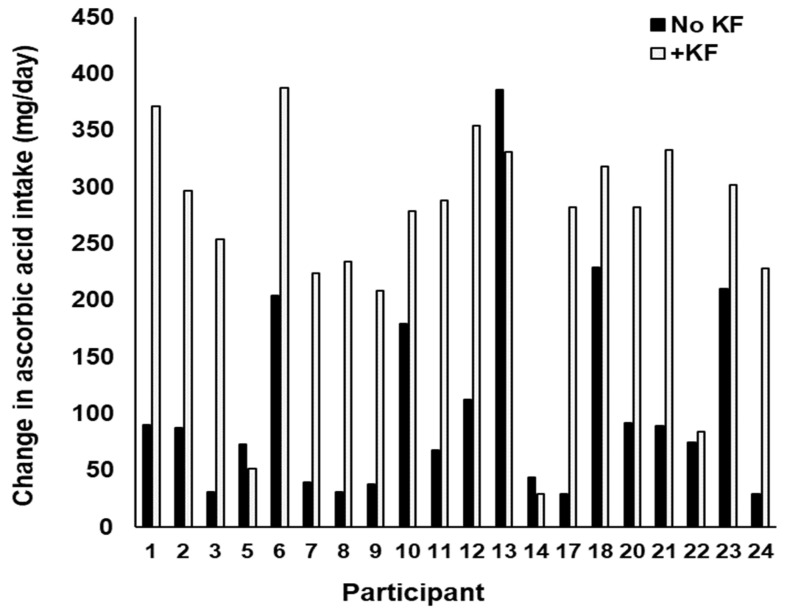
Intakes of vitamin C during the kiwifruit intervention and control periods for each participant based on 3-day food diaries.

**Figure A4:**
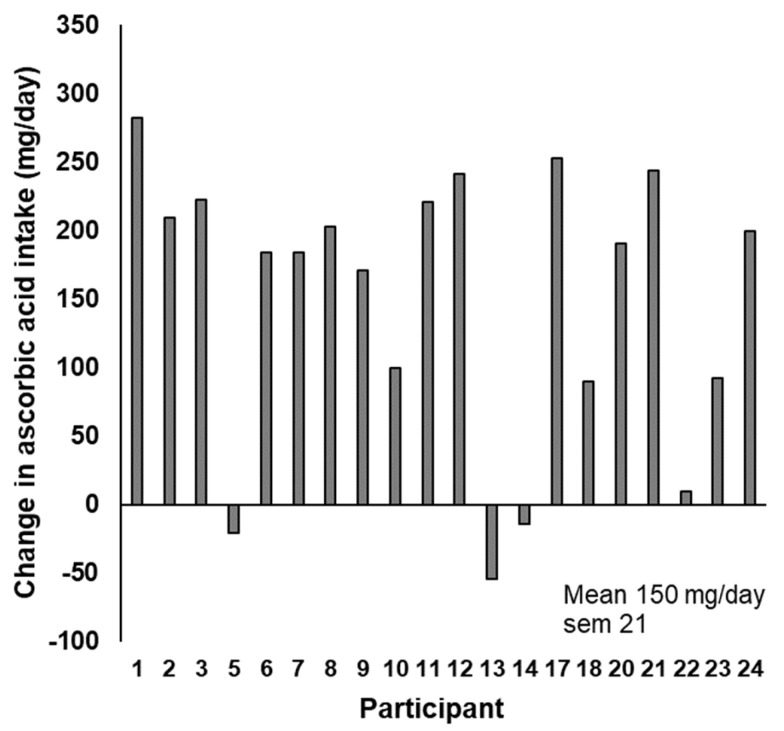
Change in daily ascorbic acid intake as a result of consuming two SunGold kiwifruit per day, compared with customary diet excluding kiwifruit, assessed for each participant who completed 3-day food diaries. Kiwifruit and control group comparison of means is in Table 2.

**Figure A5:**
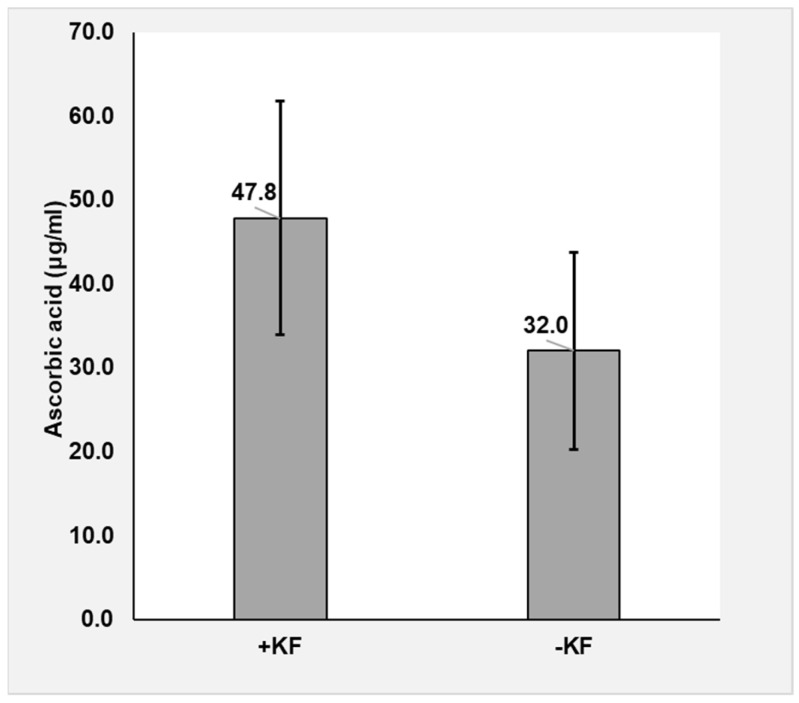
Urinary ascorbic acid concentration at end of kiwifruit intervention (+KF) and control (−KF) periods of the trial were significantly different (*p* = 0.033) (Means ± sem).

**Figure A6:**
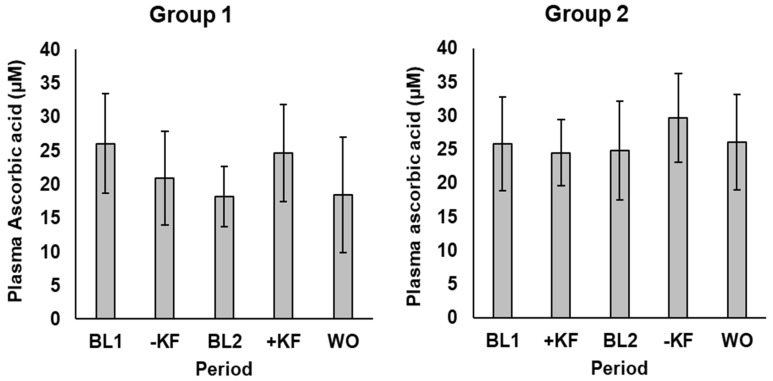
Plasma ascorbic acid concentrations at the end of no kiwifruit (−KF) and kiwifruit (+KF) interventions. BL = baseline, BL2 = baseline during crossover, WO = washout. Corresponding to periods A, B, C, D, and E in Figure 1. There were no statistically significant differences (*p* > 0.05) between treatments. (Means ± SD).

The estimated intakes of vitamin C were much higher during the kiwifruit intervention periods than during the control periods for most (17/20) participants (Figure 2, Figure A4) (Intervention, 256 ± 19; Control 106 ± 29 (Means ± sem)) who completed food diaries. Increased ascorbic acid intakes were also reflected in greater average urinary concentration of vitamin C in samples from the intervention versus control periods (Intervention, 47.8 ± 13; Control 32 ± 14 (Means ± sem)) (Table 1, Figure A5). Urinary vitamin C was significantly higher in the kiwifruit phase than the no kiwifruit phase when the baseline (starting) value was treated as a covariate. The dietary treatment had no effect on plasma ascorbic acid. (Figure A6).

#### 3.1.2. Anthropometric Measures

There was no evidence of weight gain induced by kiwifruit in the diet. Thus, if any increase in adiposity had resulted from fructose-induced lipogenesis [29], it was not sufficient to significantly affect BMI (Table 2).

#### 3.1.3. Cardiovascular Variables

The systolic blood pressure of the kiwifruit intervention group was significantly (*p* = 0.003) higher than that of the no-kiwifruit group, by 3.4%, at the end of the intervention. However, there was no significant change in systolic blood pressure between the start and finish of either the intervention or control periods (Table 2).

#### 3.1.4. Metabolic Biomarkers

Fasting blood glucose and HbA1c concentrations in the blood did not indicate any change in glycaemic control (Table 2). The time available for raised blood glucose to be reflected in HbA1c was less than the recommended 3-month period, but detectable change in HbA1c was nonetheless dependent on the amount of glycation occurring, and fructose has a much higher glycation potency than glucose [5].

However, the stability of plasma uric acid concentrations and the lack of difference between the kiwifruit and control groups in plasma uric acid suggest that fructose concentrations were not high enough for an increase in fructose metabolism due to kiwifruit ingestion to significantly raise the need for AMP (adenosine monophosphate) disposal through purine metabolism.

#### 3.1.5. Hormones and Peptides

Kiwifruit ingestion had no significant effect on IL-6 and CRP (Table 2), suggesting the inflammatory status of participants was unchanged by kiwifruit ingestion. These results are consistent with the lack of change in HbA1c, in so far as advanced glycation end products are formed by reactions with both glucose and fructose and are also thought to activate an inflammatory response through RAGE (Figure A1).

#### 3.1.6. Plasma Lipids

There were no significant changes in plasma lipids caused by kiwifruit ingestion (Table 2) despite the duration of the trial being ample for changes in blood lipids to appear. Therefore, any increase in fructose-induced hepatic lipogenesis caused by kiwifruit sugars was not enough to appreciably affect blood lipids and was, therefore, unlikely to affect glucose tolerance through blood lipid induced insulin resistance, which is consistent with other biomarker measurements in this study.

#### 3.1.7. Dietary Fibre and Plasma Short-Chain Fatty Acids

**Figure A7:**
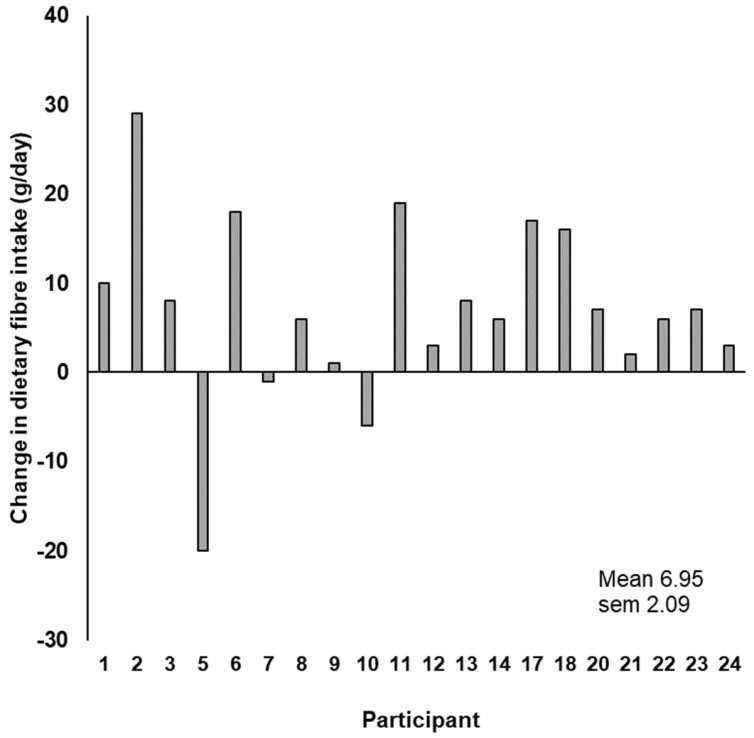
Change in daily dietary fibre intake as a result of consuming two SunGold kiwifruit per day, compared with customary diet with kiwifruit excluded, assessed from all participants who completed 3-day food diaries. Mean daily dietary fibre intake was 32 ± 2.3 g/day.

Analysis of the food diaries suggest that at times when two SunGold per day were consumed dietary fibre intakes were raised over the control period intakes for most (17/20) participants (Figure A7). On average, dietary fibre intakes were greater during the intervention than control periods by 6.95 ± 2.1 g/day. Dietary fibre was significantly higher in the kiwifruit phase than the no kiwifruit phase when the baseline (starting) value was treated as a covariate, and near-significantly higher when the change from baseline was analysed (Table 3).

**Figure A8:**
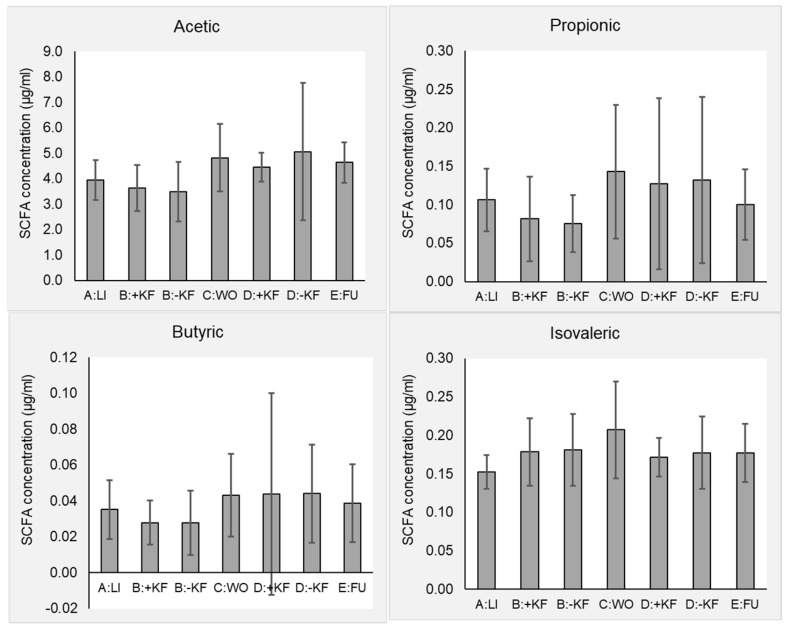
Plasma short-chain fatty acids (SCFA) at the end of different stages of long-term crossover study: A: LI = lead-in; B: +KF = first kiwifruit intervention arm; B: −KF = first control group arm; C: WO = wash out at crossover, all participants; D: +KF = second kiwifruit intervention arm; D: −KF = second control group arm; E: FU = post-trial follow up with all participants (Means ± SD). Differences between treatments were not statistically significant (*p* > 0.05).

**Figure A9:**
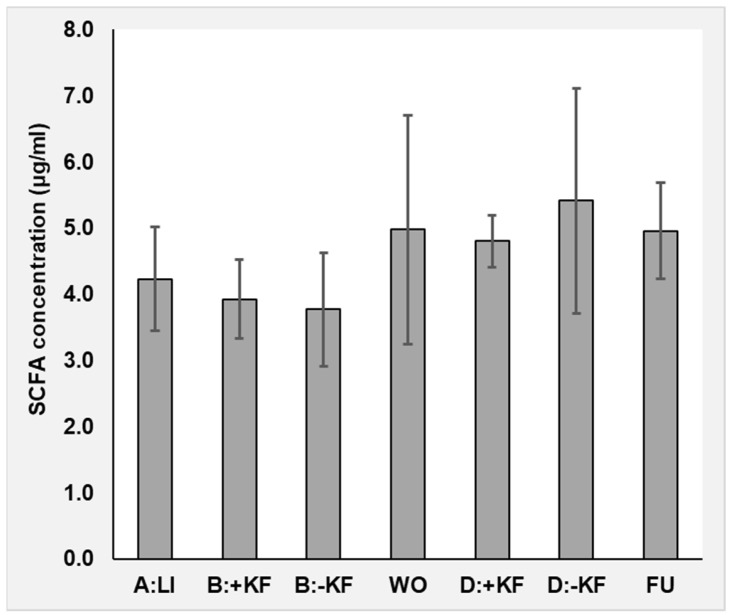
Total plasma short-chain fatty acids (SCFA) measured as the sum of acetic, propionic, butyric and isovaleric acids (Figure A5) (Means ± SD).

There were systematic differences between the treatments in plasma SCFA concentrations (Table 3); however, these were mainly between the first and second intervention periods, rather than between the kiwifruit intervention and control arms within comparison periods B and D (Figure A8 and Figure A9).

## 4. Discussion

The analysis of the range of biomarkers and anthropometric characteristics measured (Figure A1, Table A1) did not reveal any pattern of change that would indicate either a significantly improved or diminished health status, both in terms of the effect of kiwifruit on metabolic health or systemic inflammation or on metabolic functioning as a whole.

Nonetheless, because kiwifruit contain components that could have had a negative or positive effect, it was important to scientifically establish that when healthy consumers choose to include kiwifruit in a mixed diet there is no deterioration in their general health, assessed by a range of criteria. The fact that positive changes in biomarkers were not detected is not surprising in a healthy population, as most physiological systems are probably functioning near their optimal homeostatic set points, and the biochemical changes achieved by reasonably altering fruit ingestion by a healthy group are likely to be small compared with those that might be shown in a nutrient-deficient group.

### 4.1. Ascorbic Acid

Because vitamin C occurs in exceptionally high concentrations in kiwifruit [2], urinary vitamin C was used as a dual marker of participant compliance and of its contribution to antioxidant potential. While the increase in vitamin C intake in the kiwifruit intervention periods estimated from the food diaries was not reflected in the plasma ascorbic acid values, which were not consistently raised by kiwifruit ingestion, the elevation in urinary ascorbic acid indicated compliance. The results also suggest that the stability of the plasma vitamin C concentrations reflects homeostatic control achieved through a combination of urinary excretion and hepatic catabolism [20], of which only the former was measured in the present study.

Both the plasma and urinary vitamin C values showed a coefficient of variation of about 30%, typical of physiological measurements. The high degree of within-group variability in urinary ascorbic acid may have been partly because the measurements were, for practical reasons, made on a single urine specimen taken at the time of blood sampling, rather than on a 24 h collection that would have allowed for daily vitamin C excretion to be calculated. Even so, the fact that, for most participants (17/20), the vitamin C intake was greater in the kiwifruit intervention compared with the control period (Figure 2) and that this was supported by the urinary Vitamin C results (Table 2), suggest that compliance with the instruction to consume 2 kiwifruit per day was generally good. The average increase in ascorbic acid intake of 150 ± 20 mg/day (mean ± sem) from consuming two SunGold kiwifruit per day indicates that ingesting one or two of the fruit per day would eliminate the need for vitamin C supplements, as the recommended daily intake for males is at least 90 mg.

### 4.2. Metabolic/Cardiovascular Variables

Use of HbA1c as a measure of exposure to glycaemia has been criticized as inaccurate. However, misleading results have been due mainly to the presence of co-morbidities, but also to vitamin C ingestion [30]. Vitamin C intakes reportedly leading to underestimation of HbA1c were 1.0 g/day, much more than in the present study, in which the kiwifruit group was estimated to consume an average of 257 mg/day (Table A1). Furthermore, in a more recent study of the effects of vitamin C on HbA1c assays, in which participants consumed a vitamin C supplement of 1 g/day, no effect of vitamin C on HbA1c measurement was detected [31]. The term of the present study (6 weeks) was half of that usually recommended for clinical use of HbA1c in diagnosis of diabetes, which limited its value as a marker of exposure to glycaemia. However, as measured HbA1c is a moving average it would have been affected had blood glucose concentrations been extremely high in the 6 weeks prior to analysis.

Although there was a small difference in systolic blood pressure between the kiwifruit and no-kiwifruit groups at the end of the intervention and control periods, a previous long-term kiwifruit intervention trial study showed the opposite trend [22], and there was no change between the start and finish of the kiwifruit intervention in the present study (Table 2). The results, and the uniformity of pulse across groups and treatments, suggest that the small difference in blood pressure at the trial end is no reason for concern, although blood pressure is a variable that could be re-examined in a future appropriately powered trial.

Creatinine concentrations remained stable, suggesting no change in kidney function or diabetic nephropathy as a result of consuming the kiwifruit. Adiponectin was also unaffected by kiwifruit ingestion, so that no adiponectin-mediated decrease in insulin resistance was likely, consistent with the observed stability in plasma glucose and insulin concentrations. There was also no evidence of fructose-induced lipogenesis [29] due to kiwifruit ingestion, or of resulting dyslipidaemia and associated insulin resistance in this study. Plasma lipids, insulin, and fasting blood glucose all remained unchanged.

The results suggest that, in a healthy population in which homeostasis is operating normally, daily ingestion of two kiwifruit is unlikely to perturb metabolic control. Consequently, down-stream biomarkers of inflammation that may have responded to hyperglycaemia and hyperglycaemia-induced oxidative stress, namely CRP and IL-6, were unchanged.

### 4.3. Short-Chain Fatty Acids

The present study did not reveal any significant effects of consuming two SunGold kiwifruit per day on plasma SCFA. However, the trial was designed to examine the long-term effects of kiwifruit consumption rather than the transient postprandial or post-ileal (colonic) effects that could be expected from a labile fibre source such as the parenchyma cell walls of kiwifruit. There is a steep negative biological gradient in SCFA concentrations after absorption from the colon [24]. Blood sampling will need to be related to the likely period of colonic fermentation and take account of the dynamics of SCFA absorption, transport, and disposal within the body to measure the likely transient influence of kiwifruit ingestion on plasma SCFA. Further studies aimed at detecting the effects of kiwifruit on plasma SCFA will require greater control over diets and fit-for-purpose experimental designs.

Another reason for not detecting a change in SCFA is that the dietary fibre complement of SunGold kiwifruit is not intrinsically very different in chemical structure from that of many other plant foods in the background diet [32], so differences in the proportions of SCFAs would be difficult to detect given the dietary background and its variability. SunGold kiwifruit is also low in dietary fibre (1.4%) so it would be unlikely to substantially increase fermentation or plasma loading of fermentation products when consumed against the 32 g/day background fibre intake estimated for participants in the present study.

The present study in, combination with studies with Asian [21] (genetically diabetes-prone [33]), and pre-diabetic [22] (metabolically diabetes-prone) participants, has further established that there is a low probability of any harm arising from the consumption of two kiwifruit daily in a mixed diet. Healthy cohorts have been shown to differ from diabetics in their response to fructose [10], and to start accumulating effects at an early age [16], so the use of a healthy cohort in the present study was justified. However, research on the effect of age on response to fruit intake would be valuable in further research designed specifically to address this question.

The lack of significant effects in the present study can be explained as the result of effect sizes being too small to reach required statistical thresholds under the experimental conditions used. The results show that if any biomarker effects were occurring, they were so small that any changes in health status that they might indicate, if they existed, would probably also be small. However, as the possibility remains that a small change in some biomarkers could be linked to physiologically significant changes or pathology, more highly powered studies with such biomarkers as primary outcomes could be indicated. A wide but not comprehensive range of biomarkers was used to look for health-related changes induced by two kiwifruit daily, and the study population was healthy and consumed a mixed diet. Ideally a larger range of biomarkers would have been used, and the study would have been more highly powered and of longer duration, had the resources been available. However, because most biomarkers are interacting parts of a metabolic/physiological system reacting to diet (Figure A1), and all variables measured had well-established links to health outcomes, the consistency in responses across all measurements supports the view that introducing two kiwifruit per day into a mixed diet of healthy people would be a benign change, without acute or substantial effects. Furthermore, beneficial changes not measured in the present study may result from improved gut function.

An obvious limitation of the present study was its reliance on food diaries, and it was unfortunate that precise measurement and control of food intakes over the 6-week study period was beyond the resources of the study. However, this is a common and unavoidable limitation of such studies. But if any dietary effects were severe enough to become apparent in an exploratory study such as the present one, they would be subjected to much-more-strictly controlled clinical experimental analysis.

Power analyses were not applied to the range of outcomes measured during planning of the study because it was exploratory research with no null hypothesis or single primary outcome. However, it was established in advance that the KF dose caused a highly significant and definite post-prandial glycaemic response with the number of subjects used in the study (Figure A2). The present study suggested that in a healthy population consuming a mixed diet, ingestion of two kiwifruit per day would be unlikely to cause changes in the measured variables large enough to warrant further detailed analysis. If the research had identified a statistically and physiologically significant change, it could be subjected to clinical analysis as the primary outcome in a subsequent trial, for which an analysis of power would be used in planning.

The intake of two kiwifruit per day in the present study has been commonly used as a reasonable customary intake in nutritional studies. However, a single SunGold kiwifruit (100 g edible portion) provides more than the twice the daily recommended dose of vitamin C. So, in light of the results presented here, showing no negative effects of daily ingestion of two kiwifruit, kiwifruit at a dose of one per day appears to be safe to rely on as a rich source of Vitamin C.

## 5. Conclusions

The present study with a healthy, predominantly Caucasian cohort has extended and consolidated the finding that, generally, including up to two kiwifruit daily in customary diets of healthy humans is unlikely to induce metabolic or inflammatory effects of fruit sugar that are large enough to be harmful.

## Figures and Tables

**Table 1:** Effects of kiwifruit on ascorbic acid intakes and concentration in urine. (**A**) Ascorbic acid intake estimated from food diaries and urine ascorbic acid concentrations. (**B**) Table of means for ascorbic acid at start and end of no-kiwifruit and plus-kiwifruit phases.

(A)										
	Difference between Start and Finish within Treatments					Values at Finish with Start as Covariate				
	Control	Kiwi	l.s.d.	F	*p*	Control	Kiwi	l.s.d.	F	*p*
Vit C intake mg/day (n = 20)	3	156	59	30.2	<0.001	107	257	46	47.7	<0.001
Square root of urinary Vit C µg/mL (n = 22)	1	3	3	1.0	0.328	3	6	2	5.3	0.033
(**B**)										
	**Control Start** **No Kiwifruit**		**Control End** **No Kiwifruit**			**Kiwifruit Start**			**Kiwifruit End**	
	Mean	SD	Mean		SD	Mean	SD		Mean	SD
Vit C intake mg/day (n = 20)	109	67	107		94	109	85		257	98
Urinary Vit C µg/mL (n = 22)	14	38	31		66	14	23		46	61

l.s.d.—least significant difference at 5% level.

**Table 2:** Mean changes in biomarkers resulting from ingesting two kiwifruit daily for 6 weeks in a cross-over intervention study (Figure 1). Statistical analysis using two analytical approaches.

	Within-Treatment Difference between Start and Finish of 6 Week Intervention					Values at Finish with Start as Covariate				
Variables	Control	Kiwi	l.s.d.	F	*p*	Control	Kiwi	l.s.d.	F	*p*
*Anthropometric*										
BMI kg/m^2^	−0.02	−0.01	0.28	0.0	0.947	26.6	26.7	0.18	1.1	0.302
Waist:hip ratio	0.0093	0.006	0.0203	0.1	0.739	0.9722	0.9725	0.0134	0.0	0.965
*Circulatory*										
Systolic BP mmHg	−0.4	3.0	4.0	3.0	0.103	127.7	132.0	2.6	12.5	0.003
Diastolic BP mmHg	−0.9	0.5	4.4	0.4	0.527	81.5	83.3	2.2	3.1	0.096
Pulse bpm	−2.6	−4.1	3.3	0.8	0.374	59.0	58.4	2.5	0.3	0.626
*Metabolic*										
HbA1c mmol/mol	1.15	0.31	1.34	1.7	0.206	32.05	31.26	0.91	3.4	0.084
Bloodglucose mmol/L	0.033	−0.080	0.186	1.7	0.216	4.258	4.164	0.209	0.9	0.356
Insulin µ/mL	0.63	−0.04	1.46	0.9	0.352	5.96	5.21	0.95	2.8	0.113
Uric acid mmol/L	−0.013	−0.015	0.022	0.0	0.843	0.347	0.345	0.014	0.1	0.756
Creatinine µmol/L	−1.0	−3.4	3.3	2.3	0.145	90.3	88.6	2.3	2.4	0.140
*Inflammatory*										
Adiponectin mg/mL	0.15	−0.25	1.92	0.2	0.664	6.52	6.54	0.68	0.0	0.961
CRP mg/mL	2.21	0.77	4.27	0.5	0.486	2.90	1.40	4.35	0.5	0.491
IL-6 pg/mL	−0.07	0.04	0.46	0.2	0.644	0.90	1.06	0.34	1.1	0.315
*Lipidaemic*										
Cholesterol mmol/L	0.11	0.09	0.36	0.0	0.893	5.01	4.99	0.36	0.0	0.893
LDL Chol mmol/L	0.09	0.15	0.24	0.3	0.618	2.75	2.79	0.22	0.2	0.677
HDL Chol mmol/L	0.026	−0.012	0.143	0.3	0.586	1.686	1.649	0.148	0.3	0.599
Triglycerides mmol/L	−0.02	−0.04	0.20	0.0	0.851	1.26	1.25	0.14	0.0	0.930

l.s.d.—least significant difference at 5% level.

**Table 3:** Dietary fibre intake and plasma short-chain fatty acid changes due to ingesting two kiwifruit per day. (**A**) Significance of differences. (**B**) Table of means for each measure at start and end of each control and intervention period.

(A)										
	Difference between Start and Finish within Treatments					Values at Finish with Start as Covariate				
	Control	Kiwi	l.s.d.	F	*p*	Control	Kiwi	l.s.d.	F	*p*
Dietary fibre intake g/day (n = 20)	−3	3	7	3.4	0.081	29	36	5	11.0	0.004
Acetic acid	−0.24	−0.16	0.94	0.0	0.865	4.29	4.02	0.62	0.8	0.373
Propionic acid	−0.026	−0.014	0.048	0.3	0.613	0.105	0.103	0.031	0.0	0.894
Butyric acid	−0.006	−0.001	0.015	0.6	0.459	0.036	0.037	0.015	0.1	0.804
Isovaleric acid	0.001	−0.005	0.022	0.3	0.567	0.181	0.174	0.014	1.1	0.322
**(B)**										
			**No Kiwifruit** **Control Start**		**No Kiwifruit** **Control End**		**Kiwifruit Start**		**Kiwifruit End**	
			**Mean**	**SD**	**Mean**	**SD**	**Mean**	**SD**	**Mean**	**SD**
Dietary fibre intake g/day (n = 20)			33	8.9	29	13.4	33	13.4	36	13.4
Acetic acid (n = 20)			4.47	1.16	4.16	1.43	4.27	0.939	4.07	0.63
Propionic acid (n = 20)			0.132	0.076	0.100	0.054	0.116	0.040	0.106	0.06
Butyric acid (n = 20)			0.041	0.018	0.035	0.018	0.037	0.018	0.036	0.03
Isovaleric acid (n = 20)			0.180	0.054	0.180	0.031	0.179	0.049	0.175	0.02

l.s.d.—least significant difference at 5% level.

## Data Availability

Data are available from the corresponding author.

## Appendix A

**Table A1:** Biomarkers related to health outcomes that could be affected by sugar components of fruit.

Biomarker	Relationship to Health Endpoint
*Anthropometric*	
BMI kg/m^2^	A measure of overweight and obesity associated with many non-communicable diseases [34].
Waist:hip ratio	Alternative measure of obesity. Central obesity is a component of metabolic syndrome and predictive of many health outcomes [35].
*Circulatory*	
Systolic BP mmHg	Risk factor for cardiovascular disease and many other circulation-related disorders [36].
Diastolic BP mmHg	An independent risk factor for cardiovascular disease [37,38].
Pulse bpm	Positively related to cardiovascular and associated diseases and to all-cause mortality [39].
*Metabolic*	
Blood glucose mmol/L	High blood glucose concentrations increase risk of numerous diseases typical of diabetic complications, by several mechanisms [13].
HbA1c mmol/mol	An indicator of the extent of glycation over an extended period and elevated risk of numerous diabetic complications resulting from high blood glucose [40,41,42].
Insulin uU/mL	High insulin is a risk factor for a large number of medical disorders [43].
Uric acid mmol/L	Fructose metabolism elevates uric acid, which may promote metabolic syndrome [16].
Creatinine µmol/L	Metabolite with diverse effects including improved glucose tolerance [44]. A marker of kidney function and diabetic nephropathy.
Adiponectin mg/mL	Leads to improved glucose tolerance and lipid metabolism [45].
*Inflammatory*	
CRP mg/mL	Widely used indicator of inflammation induced by IL-6 and predicting diabetic complications [46] and cardiovascular disease [47].
IL-6 pg/mL	Inflammatory cytokine induced by hyperglycaemia and implicated in insulin resistance and ongoing progression to diabetes and its complications [46,48].
*Lipidaemic*	
Cholesterol mmol/L LDL Chol mmol/L HDL Chol mmol/L Triglycerides mmol/L	Dyslipidaemia combines with hyperglycaemia, hypertension, and duration in emergence of pathophysiology underlying diabetic kidney disease, diabetic retinopathy, diabetic neuropathy, and cardiovascular disease [49]. Diabetic dyslipidaemia is characterised by elevated fasting and postprandial triglycerides, low HDL-cholesterol, and elevated LDL-cholesterol particles, which represent the major link between diabetes and the increased cardiovascular risk of diabetic patients [50].

Abbreviations: BMI, body mass index; BP, blood pressure; HbA1c, glycated haemoglobin; CRP, C-reactive protein; IL-6, interleukin-6; LDL, low density lipoprotein; HDL, high density lipoprotein.

**Table A2:** Characteristics of study population.

	Average	SD	Range
Height (m)	1.8	0.1	1.67–1.87
Weight (kg)	83.8	13.7	66–131
BMI	26.2	3.3	20.9–37.5
Systolic BP	131.2	13.4	100–161
Diastolic BP	84.2	8.9	70–98
Pulse	63.0	10.4	45–87
HbA1c	29.3	3.3	23–36
Glucose	4.42	0.39	3.65–5.15
Ethnicity:	Trial start	Trial finish	
Caucasian	17	15	
Chinese	2	1	
Vietnamese	2	1	
Indian	3	3	

**Table A3:** Table of means and standard deviations for each variable measured at the start and end of the kiwifruit intervention and control phases of the trial.

	Control Start		Control End		Kiwifruit Start		Kiwifruit End	
	(n = 22)		(n = 21)		(n = 22)		(n = 21)	
Variable	Mean	SD	Mean	SD	Mean	SD	Mean	SD
*Anthropometric*								
BMI kg/m^2^	26.6	3.10	26.6	3.16	26.7	3.05	26.7	3.21
Waist:hips ratio	0.96	0.04	0.97	0.05	0.97	0.03	0.97	0.03
*Circulatory*								
Systolic BP mmHg	128	10.8	128	9.62	130	8.44	133	8.25
Diastolic BP mmHg	81.9	8.91	82.2	8.25	83.2	7.97	83.5	7.79
Pulse bpm	61.4	9.38	59.4	10.5	62.3	9.38	57.8	9.17
*Metabolic*								
HbA1c mmol/mol	30.8	3.00	32.1	3.35	31.1	3.99	31.5	3.25
Blood glucose mmol/L	4.19	0.35	4.18	0.39	4.29	0.43	4.26	0.43
Insulin uU/mL	5.23	3.47	6.03	2.84	5.29	2.49	5.33	2.57
Creatinine µmol/L	91.6	13.1	90.3	13.3	92.2	12.2	88.6	13.3
*Inflammatory*								
Adiponectin mg/mL	6.31	3.42	6.57	3.02	6.81	5.07	6.38	2.61
Uric acid mmol/L	0.36	0.05	0.34	0.05	0.36	0.06	0.35	0.05
CRP mg/mL	0.77	1.13	3.15	8.57	0.61	0.94	1.57	4.08
IL 6 pg/mL	0.95	0.66	0.92	0.55	1.02	0.89	1.07	1.05
*Lipidaemic*								
Cholesterol mmol/L	4.87	1.17	5.05	0.96	4.89	1.13	5.01	1.19
HDL Chol mmol/L	1.65	0.35	1.67	0.33	1.66	0.39	1.66	0.33
LDL Chol mmol/L	2.65	0.98	2.79	0.87	2.64	0.94	2.79	0.96
Triglycerides mmol/L	1.29	0.80	1.3	0.60	1.31	0.70	1.24	0.69

## References

[1] Wang D.D., Li Y.P., Bhupathiraju S.N., Rosner B.A., Sun Q., Giovannucci E.L., Rimm E.B., Manson J.E., Willett W.C., Stampfer M.J. (2021). Fruit and Vegetable Intake and Mortality Results from 2 Prospective Cohort Studies of US Men and Women and a Meta-Analysis of 26 Cohort Studies. Circulation.

[2] Richardson D.P., Ansell J., Drummond L.N. (2018). The nutritional and health attributes of kiwifruit: A review. Eur. J. Nutr..

[3] Sun H., Saeedi P., Karuranga S., Pinkepank M., Ogurtsova K., Duncan B.B., Stein C., Basit A., Chan J.C.N., Mbanya J.C. (2022). IDF Diabetes Atlas: Global, regional and country-level diabetes prevalence estimates for 2021 and projections for 2045. Diabetes Res. Clin. Pract..

[4] Mengstie M.A., Abebe E.C., Teklemariam A.B., Mulu A.T., Agidew M.M., Azezew M.T., Zewde E.A., Teshome A.A. (2022). Endogenous advanced glycation end products in the pathogenesis of chronic diabetic complications. Front. Mol. Biosci..

[5] Gugliucci A. (2017). Formation of Fructose-Mediated Advanced Glycation End Products and Their Roles in Metabolic and Inflammatory Diseases. Adv. Nutr..

[6] Wang X., Ouyang Y.Y., Liu J., Zhu M.M., Zhao G., Bao W., Hu F.B. (2014). Fruit and vegetable consumption and mortality from all causes, cardiovascular disease, and cancer: Systematic review and dose-response meta-analysis of prospective cohort studies. BMJ-Br. Med. J..

[7] Stanhope K.L., Medici V., Bremer A.A., Lee V., Lam H.D., Nunez M.V., Chen G.X., Keim N.L., Havel P.J. (2015). A dose-response study of consuming high-fructose corn syrup-sweetened beverages on lipid/lipoprotein risk factors for cardiovascular disease in young adults. Am. J. Clin. Nutr..

[8] Laughlin M.R. (2014). Normal Roles for Dietary Fructose in Carbohydrate Metabolism. Nutrients.

[9] Mishra S., McLaughlin A., Monro J. (2023). Food Order and Timing Effects on Glycaemic and Satiety Responses to Partial Fruit-for-Cereal Carbohydrate Exchange: A Randomized Cross-Over Human Intervention Study. Nutrients.

[10] Macedo R.C.O., Vieira A.F., Moritz C.E.J., Reischak-Oliveira A. (2018). Effects of fructose consumption on postprandial TAG: An update on systematic reviews with meta-analysis. Br. J. Nutr..

[11] Hieronimus B., Medici V., Bremer A.A., Lee V., Nunez M.V., Sigala D.M., Keim N.L., Havel P.J., Stanhope K.L. (2020). Synergistic effects of fructose and glucose on lipoprotein risk factors for cardiovascular disease in young adults. Metab.-Clin. Exp..

[12] Mukherjee N., Lin L., Contreras C.J., Templin A.T. (2021). beta-Cell Death in Diabetes: Past Discoveries, Present Understanding, and Potential Future Advances. Metabolites.

[13] Brownlee M. (2001). Biochemistry and molecular cell biology of diabetic complications. Nature.

[14] Helsley R.N., Moreau F., Gupta M.K., Radulescu A., DeBosch B., Softic S. (2020). Tissue-Specific Fructose Metabolism in Obesity and Diabetes. Curr. Diabetes Rep..

[15] Stanhope K.L., Schwarz J.M., Keim N.L., Griffen S.C., Bremer A.A., Graham J.L., Hatcher B., Cox C.L., Dyachenko A., Zhang W. (2009). Consuming fructose-sweetened, not glucose-sweetened, beverages increases visceral adiposity and lipids and decreases insulin sensitivity in overweight/obese humans. J. Clin. Investig..

[16] Russo E., Leoncini G., Esposito P., Garibotto G., Pontremoli R., Viazzi F. (2020). Fructose and Uric Acid: Major Mediators of Cardiovascular Disease Risk Starting at Pediatric Age. Int. J. Mol. Sci..

[17] Monro J., Mishra S., Stoklosinski H., Bentley-Hewitt K., Hedderley D., Dinnan H., Martell S. (2022). Dietary Fibre and Organic Acids in Kiwifruit Suppress Glycaemic Response Equally by Delaying Absorption-A Randomised Crossover Human Trial with Parallel Analysis of C-13-Acetate Uptake. Nutrients.

[18] Jideani A.I.O., Silungwe H., Takalani T., Omolola A.O., Udeh H.O., Anyasi T.A. (2021). Antioxidant-rich natural fruit and vegetable products and human health. Int. J. Food Prop..

[19] Salamone D., Rivellese A.A., Vetrani C. (2021). The relationship between gut microbiota, short-chain fatty acids and type 2 diabetes mellitus: The possible role of dietary fibre. Acta Diabetol..

[20] Smirnoff N. (2018). Ascorbic acid metabolism and functions: A comparison of plants and mammals. Free Radic. Biol. Med..

[21] Monro J., Lubransky A., Mishra S., Haszard J., Venn B. (2022). Metabolic and Blood Pressure Effects of Consuming Two Kiwifruit Daily for 7 Weeks: A Randomised Controlled Trial. Nutrients.

[22] Mishra S., Bentley-Hewitt K., Lubransky A., Venn B., Hedderley D., Dinnan H., Martell S., Haszard J., Monro J. (2022). Metabolic, anthropometric and blood pressure effects of adding two kiwifruit or bottled water into the diets of people with pre-diabetes: A randomised, parallel group, intervention study. Recent Prog. Nutr..

[23] Monro J.A., Mishra S. (2010). Digestion-resistant remnants of vegetable vascular and parenchyma tissues differ in their effects in the large bowel of rats. Food Dig..

[24] Morrison D.J., Preston T. (2016). Formation of short chain fatty acids by the gut microbiota and their impact on human metabolism. Gut Microbes.

[25] Fu L.M., Zhang G.B., Qian S.S., Zhang Q., Tan M.M. (2022). Associations between dietary fiber intake and cardiovascular risk factors: An umbrella review of meta-analyses of randomized controlled trials. Front. Nutr..

[26] Natto Z.S., Yaghmoor W., Alshaeri H.K., Van Dyke T.E. (2019). Omega-3 Fatty Acids Effects on Inflammatory Biomarkers and Lipid Profiles among Diabetic and Cardiovascular Disease Patients: A Systematic Review and Meta-Analysis. Sci. Rep..

[27] Gallagher C., Keogh J.B., Pedersen E., Clifton P.M. (2016). Fructose acute effects on glucose, insulin, and triglyceride after a solid meal compared with sucralose and sucrose in a randomized crossover study. Am. J. Clin. Nutr..

[28] Moreau N.M., Delepee R., Maume D., Le Bizec B., Nguyen P.G., Champ M.M., Martin L.J., Dumon H.J. (2004). Rapid measurement of C-13-enrichment of acetic, propionic and butyric acids in plasma with solid phase microextraction coupled to gas chromatography-mass spectrometry. Anal. Chim. Acta.

[29] Ter Horst K.W., Serlie M.J. (2017). Fructose Consumption, Lipogenesis, and Non-Alcoholic Fatty Liver Disease. Nutrients.

[30] Radin M.S. (2014). Pitfalls in Hemoglobin A1c Measurement: When Results may be Misleading. J. Gen. Intern. Med..

[31] Camargo J.L., Stifft J., Gross J.L. (2006). The effect of aspirin and vitamins C and E on HbA_1c_ assays. Clin. Chim. Acta.

[32] Sims I.M., Monro J.A. (2013). Fiber: Composition, structures, and functional properties. Adv. Food Nutr. Res..

[33] Ramachandran A., Ma R.C.W., Snehalatha C. (2010). Diabetes in Asia. Lancet.

[34] Kivimaki M., Strandberg T., Pentti J., Nyberg S.T., Frank P., Jokela M., Ervasti J., Suominen S.B., Vahtera J., Sipila P.N. (2022). Body-mass index and risk of obesity-related complex multimorbidity: An observational multicohort study. Lancet Diabetes Endocrinol..

[35] Dixon J.B. (2010). The effect of obesity on health outcomes. Mol. Cell. Endocrinol..

[36] Zhou B., Perel P., Mensah G.A., Ezzati M. (2021). Global epidemiology, health burden and effective interventions for elevated blood pressure and hypertension. Nat. Rev. Cardiol..

[37] Fernandez-Ruiz I. (2019). Systolic and diastolic hypertension independently predict CVD risk. Nat. Rev. Cardiol..

[38] Flint A.C., Conell C., Ren X.S., Banki N.M., Chan S.L., Rao V.A., Melles R.B., Bhatt D.L. (2019). Effect of Systolic and Diastolic Blood Pressure on Cardiovascular Outcomes. N. Engl. J. Med..

[39] Aune D., Sen A., o’Hartaigh B., Janszky I., Romundstad P.R., Tonstad S., Vatten L.J. (2017). Resting heart rate and the risk of cardiovascular disease, total cancer, and all-cause mortality—A systematic review and dose-response meta-analysis of prospective studies. Nutr. Metab. Cardiovasc. Dis..

[40] Lee S., Liu T., Zhou J.D., Zhang Q.P., Wong W.T., Tse G. (2021). Predictions of diabetes complications and mortality using hba1c variability: A 10-year observational cohort study. Acta Diabetol..

[41] Skrha J., Soupal J., Skrha J., Prazny M. (2016). Glucose variability, HbA1c and microvascular complications. Rev. Endocr. Metab. Disord..

[42] Tavakoli M., Ishibashi F. (2019). Impact of normalised HbA1c on diabetic neuropathy and other microvascular complications in Type 2 diabetes. Diabet. Med..

[43] Kolb H., Kempf K., Rohling M., Martin S. (2020). Insulin: Too much of a good thing is bad. BMC Med..

[44] Kreider R.B., Stout J.R. (2021). Creatine in Health and Disease. Nutrients.

[45] Yanai H., Yoshida H. (2019). Beneficial Effects of Adiponectin on Glucose and Lipid Metabolism and Atherosclerotic Progression: Mechanisms and Perspectives. Int. J. Mol. Sci..

[46] Stanimirovic J., Radovanovic J., Banjac K., Obradovic M., Essack M., Zafirovic S., Gluvic Z., Gojobori T., Isenovic E.R. (2022). Role of C-Reactive Protein in Diabetic Inflammation. Mediat. Inflamm..

[47] Ridker P.M., MacFadyen J.G., Glynn R.J., Bradwin G., Hasan A.A., Rifai N. (2020). Comparison of interleukin-6, C-reactive protein, and low-density lipoprotein cholesterol as biomarkers of residual risk in contemporary practice: Secondary analyses from the Cardiovascular Inflammation Reduction Trial. Eur. Heart J..

[48] Kreiner F.F., Kraaijenhof J.M., von Herrath M., Hovingh G.K.K., von Scholten B.J. (2022). Interleukin 6 in diabetes, chronic kidney disease, and cardiovascular disease: Mechanisms and therapeutic perspectives. Expert Rev. Clin. Immunol..

[49] Eid S., Sas K.M., Abcouwer S.F., Feldman E.L., Gardner T.W., Pennathur S., Fort P.E. (2019). New insights into the mechanisms of diabetic complications: Role of lipids and lipid metabolism. Diabetologia.

[50] Wu L.Y., Parhofer K.G. (2014). Diabetic dyslipidemia. Metab.-Clin. Exp..

